# AAV-Based Bright and Sparse Labeling of Versatile Neurons Adaptable in Cre-Dependent Genetic Backgrounds

**DOI:** 10.1523/ENEURO.0314-25.2026

**Published:** 2026-03-25

**Authors:** Satoshi Kamijo, Kazutaka Ikeda, Hideki Miwa

**Affiliations:** ^1^Department of Neuropsychopharmacology, National Institute of Mental Health, National Center of Neurology and Psychiatry, Kodaira, Tokyo 187-8551, Japan; ^2^Department of Physiology, National Defense Medical College, Tokorozawa, Saitama 359-8513, Japan; ^3^Tokyo Metropolitan Institute of Medical Science, Setagaya, Tokyo 156-8506, Japan

**Keywords:** adeno-associated virus, morphological analysis, Purkinje cell, recombinase, sparse labeling, Tet system

## Abstract

Sparse labeling techniques are essential for morphological analysis of the central nervous system. Various sophisticated strategies have been developed, but conventional methods, such as Golgi–Cox staining and biocytin injection, remain widely used. Recent advances in adeno-associated virus (AAV) technology have enabled sparse neuronal labeling in rodents, either through postnatal AAV injection or by coinjecting a dilute Cre recombinase-expressing driver with a Cre-dependent amplifier. However, postnatal AAV injection requires prior preparation, and Cre-dependent systems are incompatible with Cre-expressing or floxed genetic backgrounds. Here, we present a Cre-orthogonal AAV–based sparse labeling method that utilizes Supernova technology in male and female mice. We employed Flpe recombinase to achieve Cre-independent labeling. A retro-orbital injection of PHP.eB AAVs failed to label neurons, whereas local injection enabled the bright and sparse labeling of multiple neuronal types, including cerebellar neurons. The labeled neurons were sufficiently bright to visualize fine structures, such as dendritic spines, without the need for immunostaining. This method is compatible with both Cre-expressing and floxed genetic backgrounds. Furthermore, we demonstrated that the labeled neurons can be manipulated by the coinjection of a Flpe-dependent third virus. This straightforward and flexible approach is applicable across various genetic backgrounds and can be readily integrated into existing experimental systems.

## Graphical abstract

**Figure:**
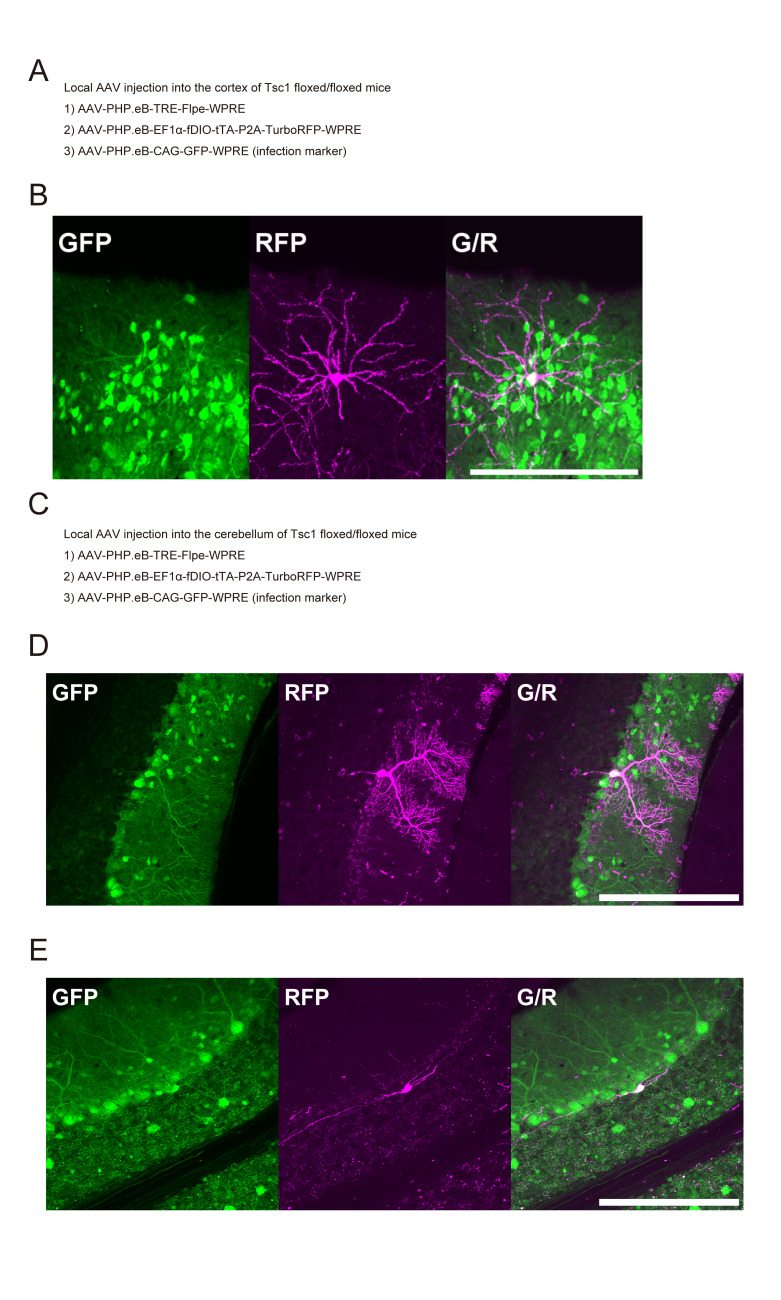
Visual Abstract

## Significance Statement

This method enables the bright and sparse labeling of diverse neuronal populations in rodents. A simple injection of two AAV vectors in the target region labels a small subset of neurons in 2–3 weeks. The labeled neurons are sufficiently bright to visualize fine subcellular structures, such as dendritic spines, without the need for immunostaining. This approach does not require complex transgenic strategies and is readily applicable to existing experimental systems. The labeling is independent of Cre recombinase, and it is broadly compatible with a wide range of Cre-expressing and floxed animals. Moreover, the labeled cells express tTA and Flpe recombinase, thereby enabling further genetic manipulation through coinfection with additional viruses.

## Introduction

The morphological analysis of neurons is an indispensable technique for understanding neural circuits. Alterations of neuronal morphology have been implicated in various neurodevelopmental and psychiatric disorders (Penzes et al., 2011). To visualize individual neurons within densely packed neural tissue, various sparse labeling methods have been developed (Jefferis and Livet, 2012; Hui et al., 2022).

Conventional techniques, such as Golgi–Cox staining and biocytin injection, are still widely used. Although they provide good contrast, Golgi–Cox staining is incompatible with immunohistochemistry, and biocytin injection is a labor-intensive process. The discovery of, and improvements in, genetically encoded fluorescent proteins significantly advanced the field (Chudakov et al., 2010). For example, Thy-1 transgenic mice have served as the gold standard for dendritic spine analysis because of their high levels of fluorescent protein expression (Feng et al., 2000).

Although other genetic systems that are specialized for morphological analysis have also been developed (Zong et al., 2005; Young et al., 2008; Veldman et al., 2020), they often require extensive breeding. Additionally, they are not easily integrated into experiments that involve animals with complex genetic backgrounds. More straightforward methods, such as in utero electroporation (IUE) and postnatal viral injection, have enabled sparse labeling in several brain regions, particularly in the cerebellum (Kaneko et al., 2011; Nishiyama et al., 2012; Takeo et al., 2015, 2021). However, these techniques must be performed during the perinatal period, making them unsuitable for ad hoc labeling in adult animals.

More recently, advances in adeno-associated virus (AAV) technology have enabled the delivery of exogenous DNA into adult neurons in a minimally invasive manner (Deverman et al., 2016; Chan et al., 2017; Bedbrook et al., 2018; Challis et al., 2022). Broadly divided, two types of AAV-based sparse neuronal labeling strategies have been reported. One involves a simple injection of diluted AAVs, and the other employs a two-component system that consists of a driver virus and an amplifier virus. In the former, low-density labeling relies on sparse infection (Chan et al., 2017), limiting the number of viral genomes per cell and often necessitating subsequent signal amplification via immunohistochemistry to visualize fine structures. In the latter, a dilute driver virus and an excess of amplifier virus are coinjected. The driver expresses a specific recombinase or tetracycline transactivator (tTA) protein, which activates the amplifier virus by recombination or through tTA binding to the tetracycline response element (TRE; Chan et al., 2017; Sakaguchi et al., 2018; Dorgans et al., 2022).

Luo and colleagues reported that Supernova system enables bright and sparse neuronal labeling (Mizuno et al., 2014; Luo et al., 2016). In this system, sparse labeling is achieved through doxycycline-independent leaky transcription from the TRE promoter and a recombinase-dependent positive feedback process. Two genetic components are introduced into target cells: a driver and an amplifier. The driver encodes a recombinase under the control of the TRE promoter, whereas the amplifier encodes tTA and a fluorescent protein under a constitutive promoter. Expression from the amplifier is initially blocked and is activated only after recombinase-mediated rearrangement by the driver.

In the initial state, all amplifiers remain inactive, and recombinase is not expressed because no TRE-binding transcriptional activator is present. However, the TRE promoter exhibits low-level leaky transcription even in the absence of such an activator. In a small subset of cells, this leakiness permits recombinase expression, leading to rearrangement of the amplifier and subsequent production of tTA and the fluorescent protein. The newly expressed tTA binds to the TRE promoter in the driver, markedly enhancing recombinase expression and thereby establishing a positive feedback loop. This self-amplifying mechanism ensures sparse yet bright labeling. Using this system, the authors showed that an injection of a Cre-dependent Supernova AAV mixture in the cerebral cortex in early postnatal mice effectively visualized cortical neuron morphology.

Building on this strategy, Lin and colleagues demonstrated that a pair of Cre-dependent Flpo-expressing driver virus and Flpo-dependent tTA and GFP-expressing virus could sparsely highlight Cre-positive neurons (Lin et al., 2018). This labeling system is optimized for Cre-expressing neurons, and its function relies on Cre recombinase. However, to fully exploit the broad availability of Cre-expressing or floxed animal lines, a Cre-independent system is needed.

In the present study, we developed a Flpe-dependent, Cre-orthogonal (i.e., Cre-independent) sparse labeling system to take full advantage of Cre-expressing and floxed animals. Although a retro-orbital injection of PHP.eB AAVs failed to label neurons, local AAV injection enabled the bright and sparse labeling of multiple neuronal types, including cerebellar neurons.

The labeled neurons were sufficiently bright to visualize fine structures without immunostaining, and the labeling density was tunable. We further demonstrated that this system is compatible with Cre-expressing and floxed genetic backgrounds and that the labeled neurons can be manipulated by coinjecting a third, Flpe-dependent virus.

## Materials and Methods

### Animals

Animals were maintained at the National Center of Neurology and Psychiatry animal facility under a 12/12 h light/dark cycle with food and water available *ad libitum*. All experiments were performed during the ZT 0–12 period.

Wild-type C57BL/6J and ICR mice were obtained from Japan SLC. The L7-Cre line was kindly provided by Dr. Kei Hori (https://www.jax.org/strain/004146, RRID: IMSR_JAX:004146; Barski et al., 2000), and the Tsc1-floxed line (https://www.jax.org/strain/005680, RRID: IMSR_JAX:005680) was generously provided by Dr. Sosuke Yagishita (Kwiatkowski et al., 2002). Both males and females were used. We did not administer doxycycline in any of the experiments.

Animals were group-housed until the injection and then single-housed afterward to prevent wound dehiscence. All animal manipulations were conducted in accordance with the guidelines for animal experiments at the National Center of Neurology and Psychiatry.

### Plasmids

The pK036.TRE-Flpe-WPRE (Supernova; RRID: Addgene_85007), pK168.AAV-EF1α-DIO-tTA-P2A-RFP-WPRE (Supernova; RRID: Addgene_85039), pK170.AAV-TRE-Cre-WPRE (Supernova; RRID: Addgene_85040), and pAAV-Ef1α-fDIO-mCherry (RRID: Addgene_114471) plasmids were obtained from Addgene. We used the TRE-tight promoter and not the latest TRE3G promoter.

To construct pAAV-TRE-Flpe-WPRE, we amplified the Flpe coding sequence from pK036.TRE-Flpe-WPRE (Supernova) and used to replace the Cre sequence in pK170.AAV-TRE-Cre-WPRE (Supernova) with the In-Fusion HD cloning kit (TaKaRa Bio).

To generate pAAV-Ef1α-fDIO-tTA-P2A-TurboRFP-WPRE, both pK168.AAV-EF1α-DIO-tTA-P2A-RFP-WPRE (Supernova) and pAAV-Ef1α-fDIO-mCherry were digested with NheI and AscI, and the mCherry fragment was replaced with tTA-P2A-RFP. All plasmids were purified using the NucleoBond Xtra Midi EF kit to minimize residual endotoxin (Macherey-Nagel).

### IUE

Pregnant ICR mice on embryonic day 15.5 (E15.5) were anesthetized with a combination of 0.23 mg/kg medetomidine, 3.00 mg/kg midazolam, and 3.75 mg/kg butorphanol. The uterus was exposed, and a DNA solution that was colored with Fast Green was injected in the lateral ventricle of the embryos using a glass capillary (Saito and Nakatsuji, 2001). Square wave pulses (five pulses, 45 V amplitude, 50 ms duration, 1 s interval) were applied to each embryo using 5-mm-diameter electrodes (CUY650P5, Nepa Gene). Electrical pulses were delivered by an electroporator (CUY21, Nepa Gene). The abdominal incision was sutured with nylon thread, and the animals were allowed to recover.

**Figure 1.**
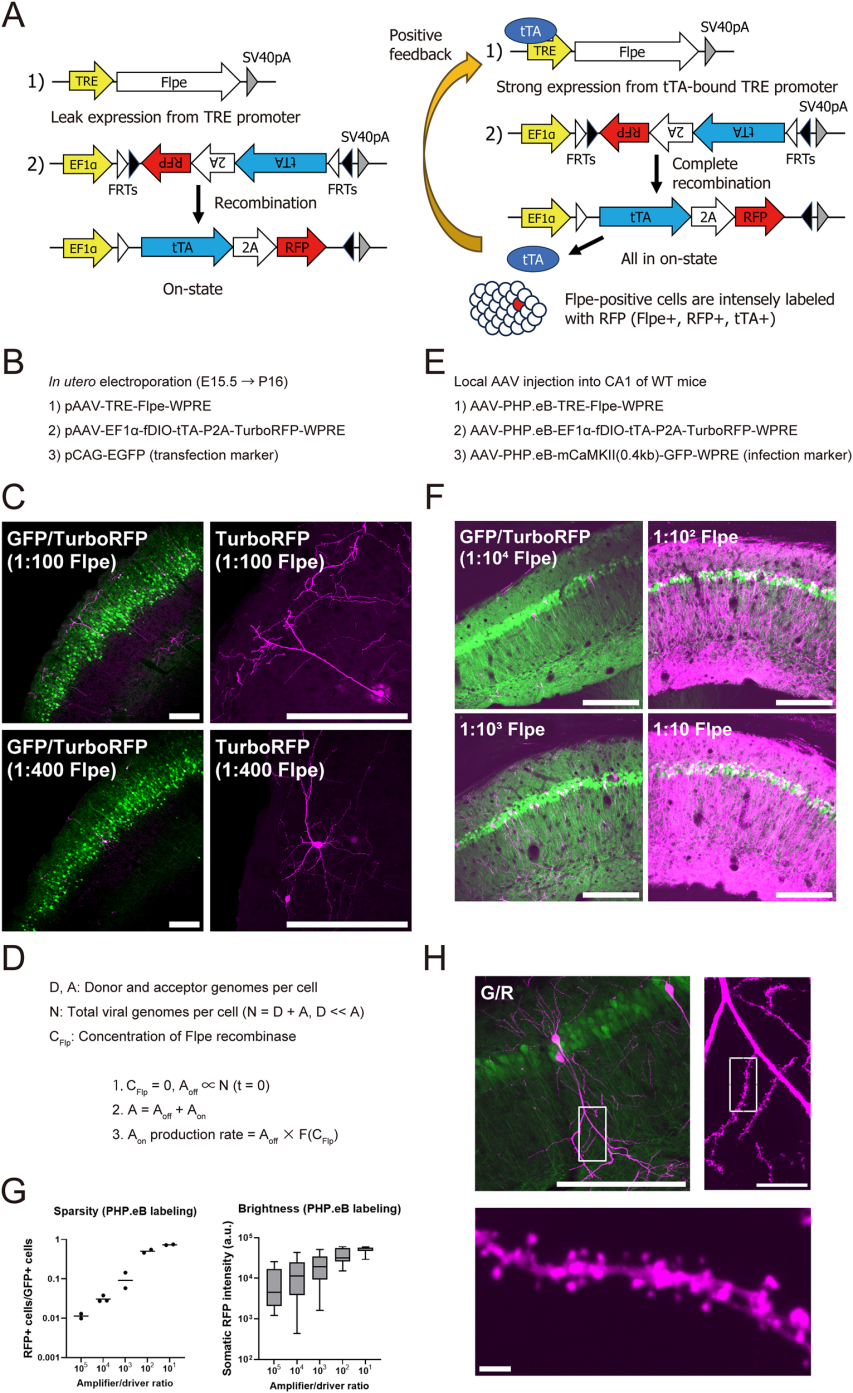
Validation of Flpe-dependent sparse labeling. ***A***, Two-component labeling system with positive feedback in the initial state (left) and during positive feedback (right). ***B***, Experimental condition of IUE. ***C***, Flpe-dependent sparse labeling of P16 mouse cortical neurons by IUE (GFP, transfection marker; TurboRFP, sparse labeling; scale bar, 200 µm). ***D***, Parameters of the model and three equations describing the labeling process are shown. ***E***, Experimental condition of the PHP.eB AAV injection in the adult mouse hippocampus. ***F***, Tunable sparse labeling with PHP.eB AAV in the hippocampal CA1 region at various driver/amplifier ratios (GFP, infection marker; TurboRFP, sparse labeling; scale bar, 200 µm). ***G***, Sparsity of cells labeled with PHP.eB virus (left, RFP+/GFP+ cells: 10^5^, 1.1%; 10^4^, 3.1%; 10^3^, 10%; 10^2^, 51%; 10^1^, 74%; geometric mean with individual data points; *N* = 2–3) and their brightness (right; box plots show the median and interquartile range, with whiskers indicating the full range; *N* = 2–3). ***H***, Close-up views of dendritic spines of CA1 neurons labeled under the 1:10^4^ condition (left, low-magnification view; scale bar, 200 µm; right, higher-magnification view of dendrites from the inset; scale bar, 20 µm; bottom, close-up view of spines from the inset; scale bar, 2 µm). See also Extended Data Figures 1-1 and 1-2.

The composition of the DNA solution in each experiment is described below, with final concentrations in parentheses. In Figure 1, *B* and *C*, the 1:100 labeling condition included pAAV-TRE-Flpe-WPRE (10 ng/µl), pAAV-Ef1α-fDIO-tTA-P2A-TurboRFP-WPRE (995 ng/µl), and pCAG-enhanced green fluorescent protein (EGFP) (995 ng/µl). The 1:400 condition used pAAV-TRE-Flpe-WPRE (2.5 ng/µl), pAAV-Ef1α-fDIO-tTA-P2A-TurboRFP-WPRE (999 ng/µl), and pCAG-EGFP (999 ng/µl).

In Extended Data Figure 1-1, the 1:200 and 1:50 labeling conditions included pAAV-TRE-Flpe-WPRE (5 or 20 ng/µl), pAAV-Ef1α-fDIO-tTA-P2A-TurboRFP-WPRE (1,000 ng/µl), and pCAG-EGFP (500 ng/µl). For Cre-dependent labeling, pK170.AAV-TRE-Cre-WPRE (Supernova; 5 or 20 ng/µl), pK168.AAV-EF1α-DIO-tTA-P2A-RFP-WPRE (Supernova; 1,000 ng/µl), and pCAG-EGFP (500 ng/µl) were used for the 1:200 and 1:50 conditions.

The animals were transcardially perfused on Postnatal Day 16.

### AAV production

Viral titers are indicated in parentheses. AAV-PHP.eB-TRE-Flpe-WPRE (1.85 × 10^13^ vg/ml), AAV-PHP.eB-EF1α-fDIO-tTA-P2A-TurboRFP-WPRE (1.33 × 10^13^ vg/ml), AAV-PHP.eB-mCaMKII(0.4 kb)-GFP-WPRE (2.44 × 10^13^ vg/ml), AAV9-TRE-Flpe-WPRE (3.62 × 10^13^ vg/ml), and AAV9-EF1α-fDIO-tTA-P2A-TurboRFP-WPRE (7.69 × 10^13^ vg/ml) viruses were prepared by the Vector Core Facility of Gunma University (Konno and Hirai, 2020).

AAV-PHP.eB-CAG-GFP-WPRE (2.58 × 10^13^ vg/ml) and AAV-PHP.eB-CAG-Cre-GFP-WPRE (2.63 × 10^13^ vg/ml) were obtained from SignaGen Laboratories. AAV-PHP.eB-nEF1α-fDIO-hM4D(G_i_)-EGFP-WPRE (1.10 × 10^13^ vg/ml) virus was purchased from BrainVTA. AAV8-EF1α-Con/Foff 2.0-EYFP virus (2.1 × 10^13^ vg/ml) was obtained from Addgene.

### Stereotaxic AAV injection

The animals were briefly anesthetized with 4% isoflurane and placed in a stereotaxic frame. Anesthesia was maintained with 2% isoflurane that was delivered in a continuous flow of oxygen. The skull was exposed, and burr holes were drilled above the injection sites. The following stereotaxic coordinates (from the bregma) were used: hippocampal injections (anterior/posterior, −1.8 mm; medial/lateral, ±1.0 mm; dorsal/ventral, −2.0 mm), frontal cortex (anterior/posterior, +1.7 mm; medial/lateral, −1.0 mm; dorsal/ventral, −2.0 mm), and cerebellum (anterior/posterior, −6.47 mm; medial/lateral, ±0.0 mm; dorsal/ventral, −2.5 mm).

The AAV solution was injected using a microsyringe (Hamilton Company) at a rate of 50 nl/min, controlled by a UMP3 injector (World Precision Instruments). The composition of the injected AAV solution in each experiment is shown below. For simplicity, AAV-PHP.eB-TRE-Flpe-WPRE and AAV-PHP.eB-Ef1α-fDIO-tTA-P2A-TurboRFP-WPRE are referred to as the driver and amplifier viruses, respectively.

In Figure 1, *E* and *F*, the amplifier (3 µl) and the infection marker AAV-PHP.eB-mCaMKII(0.4 kb)-GFP-WPRE (0.3 µl) were mixed with various dilutions of the driver virus (3 µl; 10×, 10^2^×, 10^3^×, or 10^4^×). A total volume of 200 nl was injected in the target site in wild-type mice. In Figure 1, *G* and *H*, and Extended Data Figure 1-1*F*,*G*, the amplifier (3 µl) and the infection marker AAV-PHP.eB-mCaMKII(0.4 kb)-GFP-WPRE (1 µl) were mixed with various dilutions of the driver virus (3 µl; 10×, 10^2^×, 10^3^×, or 10^4^×, or 10^5^×). A total volume of 250 nl was injected in the target site in wild-type mice. For AAV9 labeling, the original stock of the driver and the amplifier was diluted 2× or 5× to match the titer of the corresponding PHP.eB solutions.

**Figure 2.**
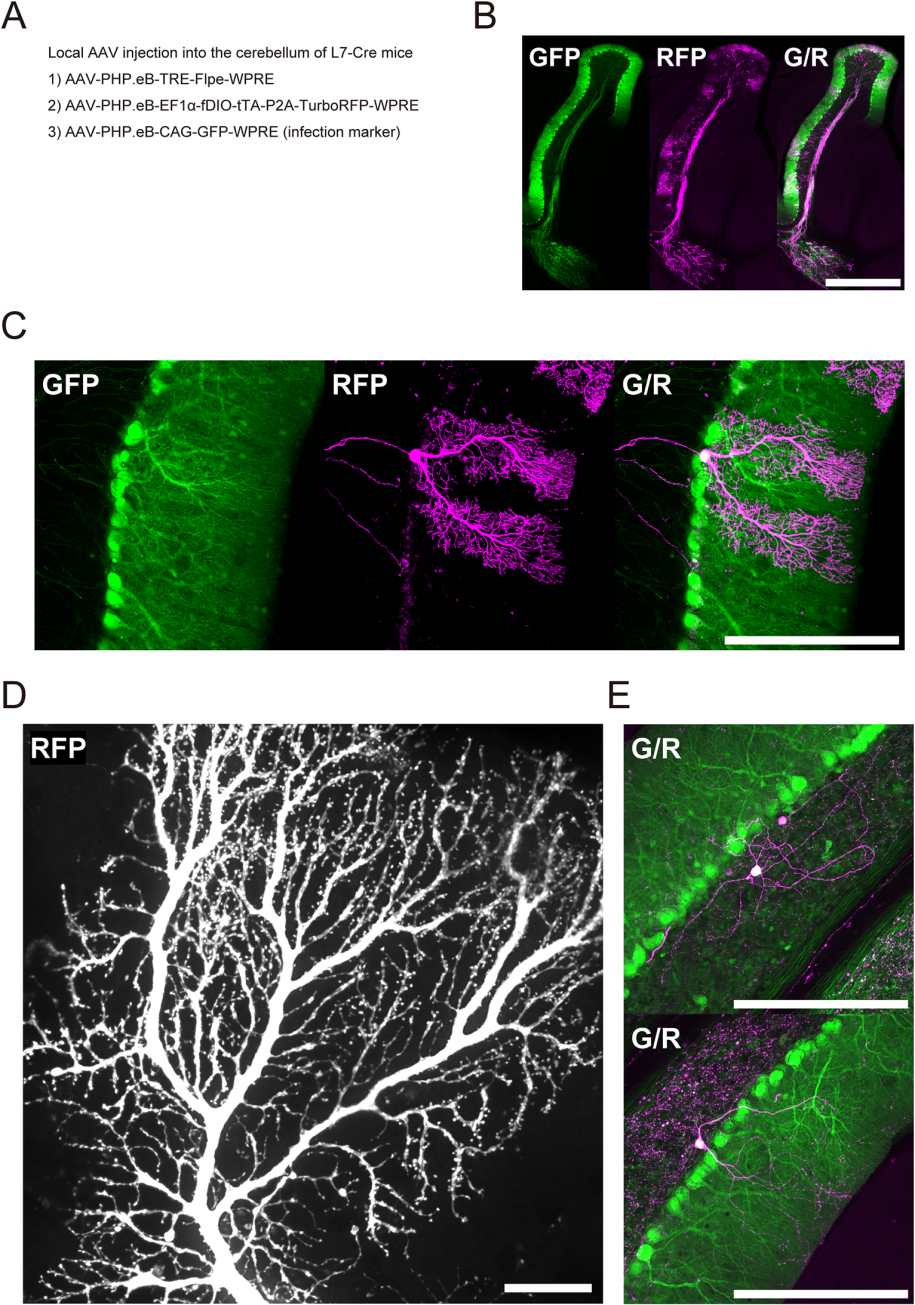
AAV-based sparse labeling of cerebellar neurons in adult L7-Cre mice. ***A***, Experimental condition of local PHP.eB AAV injection in the cerebellum in adult L7-Cre mice. ***B***, Brightly and sparsely labeled Purkinje cells 21 d after injection at a driver/amplifier ratio of 1:30 (GFP, infection marker; TurboRFP, sparse labeling; scale bar, 200 µm). ***C***, Dendritic arborization of a labeled Purkinje cell (scale bar, 200 µm). ***D***, Representative dendritic spines from a separate neuron (native TurboRFP signal; scale bar, 20 µm). ***E***, Labeled granular layer interneurons at a driver/amplifier ratio of 1:30 (scale bar, 200 µm). See also Extended Data Figure 2-1.

In Figure 2, the amplifier (3 µl), a 2× diluted AAV-PHP.eB-CAG-GFP-WPRE infection marker (1 µl) and a 10× diluted driver virus (1 µl) were mixed. A total of 250 nl was injected in the target site in L7-Cre mice. In Extended Data Figure 2-1, the same solution composition and injection volume were used as in Figure 2, except for panel *B*, and the injections were performed in Tsc1 floxed/floxed mice. In panel *B*, the driver virus was 100× diluted.

**Figure 3.**
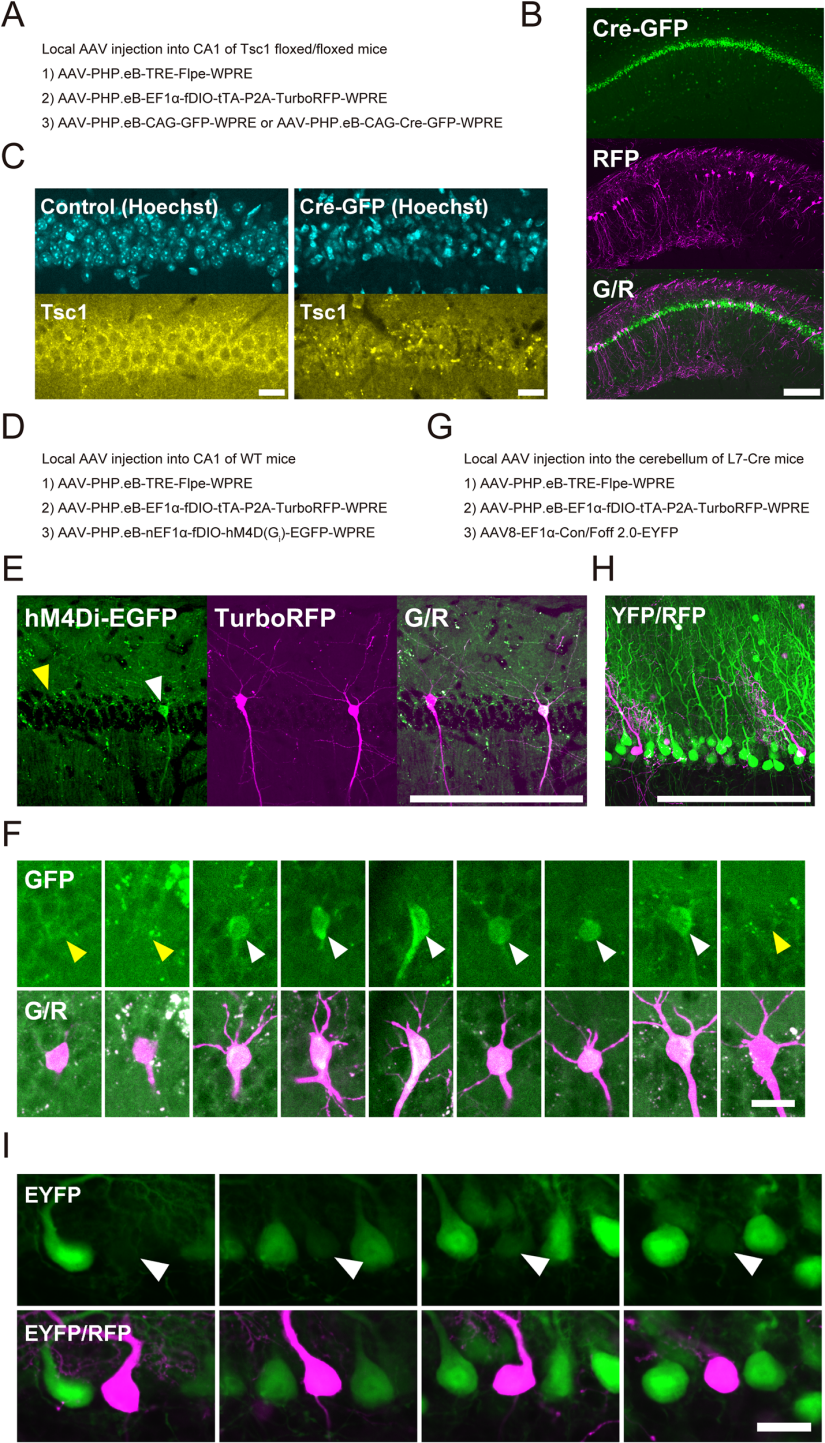
Applications of Cre-orthogonal sparse labeling. ***A***, Experimental condition of local PHP.eB AAV injection in the hippocampus in adult Tsc1 floxed/floxed mice. ***B***, Sparsely labeled hippocampal CA1 cells with Cre-GFP expression 21 d after injection at a driver/amplifier ratio of 1:500 (Cre-GFP, coinjected infection marker; TurboRFP, sparse labeling; scale bar, 200 µm). ***C***, Diminished Tsc1 immunofluorescence in Cre-expressing neurons compared with contralateral cells (Hoechst, nuclear stain; Tsc1, anti-Tsc1 immunofluorescence; scale bar, 20 µm). ***D***, Experimental condition of local PHP.eB AAV injection with a Flpe-dependent virus in the hippocampus in adult wild-type mice. ***E***, hM4Di-EGFP expression in sparsely labeled cells 21 d after injection at a driver/amplifier ratio of 1:500 (hM4Di-EGFP, expressed from a coinjected virus in a Flpe-dependent manner; TurboRFP, sparse labeling; scale bar, 200 µm). Yellow and white arrowheads indicate EGFP-negative and positive neurons, respectively. ***F***, Representative images of individual pyramidal cells are shown (GFP, Flpe-dependent expression driven by the third virus; G/R, merged GFP and RFP channels showing sparse labeling; scale bar, 20 µm). White and yellow arrowheads indicate GFP-positive and GFP-negative somata, respectively. Data were obtained from three animals (*N* = 3). The numbers of sections analyzed per animal were 3, 4, and 13, yielding 5, 5, and 16 RFP-positive cells and 2, 3, and 13 GFP-positive cells, respectively. In the same sections, 519, 765, and 1,807 Hoechst-stained nuclei were counted in the respective animals. ***G***, Experimental condition of local AAV injection with a Con/Foff 2.0-EYFP virus in the cerebellum of adult L7-Cre mice. ***H***, EYFP and TurboRFP expression in cerebellar neurons 21 d after injection at a driver/amplifier ratio of 1:30. ***I***, Representative images of individual Purkinje cells are shown (EYFP, Cre-dependent EYFP expression in the absence of Flpe; EYFP/RFP, merged EYFP and RFP channels showing sparse labeling; scale bar, 20 µm). Arrowheads indicate RFP-positive Purkinje cell somata. Data were obtained from two animals (*N* = 2). The numbers of sections analyzed per animal were five and three, yielding 6 and 5 RFP-positive cells and 161 and 117 EYFP-positive cells, respectively.

In Figure 3*A*–*C*, the amplifier (6 µl) was mixed with the 167× diluted driver virus (2 µl), and either AAV-PHP.eB-CAG-GFP-WPRE (2 µl) or AAV-PHP.eB-CAG-Cre-GFP-WPRE (2 µl) was subsequently added. A total of 250 nl was injected in each side of the hippocampus in Tsc1 floxed/floxed mice.

In Figure 3*D*–*F*, the amplifier (6 µl) was mixed with the 167× diluted driver virus (2 µl), and AAV-PHP.eB-nEF1α-fDIO-hM4D(G_i_)-EGFP-WPRE (6 µl) was added. A total volume of 250 nl was delivered to the hippocampus in wild-type mice.

In Figure 3*G*–*I*, the amplifier (6 µl) was mixed with the 10× diluted driver virus (2 µl), and AAV8-EF1α-Con/Foff 2.0-EYFP virus (2 µl) was added. A total volume of 250 nl was delivered to the cerebellum in L7-Cre mice.

The injected mice were transcardially perfused either 14 or 21 d after the injection.

### Retro-orbital AAV injection

The animals were briefly anesthetized with 4% isoflurane, and AAV solutions were injected in the retro-orbital sinus using a 27 gauge needle that was attached to a 1 ml syringe (Yardeni et al., 2011). The composition of the AAV solution in each experiment is described below.

In Extended Data Figure 1-2, the amplifier virus (70 µl), 1,000× diluted driver virus (7 µl), and AAV-PHP.eB-mCaMKII (0.4 kb)-GFP-WPRE (35 µl) were mixed and further diluted with saline to a final volume of 700 µl. A total of 200 µl of solution was injected for the 1: 10^4^ labeling experiment. For the 1:15 and 1:3 labeling conditions, the amplifier virus (60 µl), AAV-PHP.eB-mCaMKII (0.4 kb)-GFP-WPRE (30 µl), and undiluted driver virus (4 or 20 µl) were mixed and further diluted with saline to a final volume of 600 µl. A total of 250 µl of solution was injected for the 1:15 and 1:3 labeling experiments. The injected animals were transcardially perfused 21 d after the injection.

### Perfusion fixation

The animals were deeply anesthetized with a mixture of 0.69 mg/kg medetomidine, 9.00 mg/kg midazolam, and 11.25 mg/kg butorphanol and transcardially perfused with phosphate-buffered saline (PBS), pH 7.4, followed by 4% paraformaldehyde in phosphate buffer. Brains were dissected and postfixed in the same fixative overnight at 4°C. After fixation, the brains were cryoprotected by sequential immersion in 15% and then 30% sucrose PBS.

### Immunohistochemistry

Cryoprotected brains were sectioned at a thickness of 50 µm. Free-floating sections were blocked for 2 h at room temperature in phosphate buffer that contained 5% normal goat serum, 1% bovine serum albumin (BSA), and 0.3% Triton X-100. The sections were then incubated overnight at 4°C with primary antibodies that were diluted in phosphate buffer that contained 1% normal goat serum, 1% BSA, and 0.3% Triton X-100.

After two washes with PBS that contained 0.3% Triton X-100, the sections were incubated for 1 h at room temperature with secondary antibodies that were prepared in the same buffer that was used for the primary antibodies. The sections were subsequently washed twice with PBS, counterstained with Hoechst 33324, and mounted using a custom-made mounting medium.

### Antibodies

The primary antibody was mouse anti-Hamartin (C-8; 1:1,000, Santa Cruz Biotechnology, catalog #sc-377386, RRID: N/A), and the secondary antibody was goat anti-mouse IgG2b that was conjugated with Alexa Fluor 633 (1:1,000, Thermo Fisher Scientific, catalog #A-21146, RRID: AB_2535782).

### Imaging

Images were acquired using a SpinSR10 super-resolution confocal microscope (Evident formerly Olympus) and a BZ-X710 fluorescence microscope (Keyence). Sagittal brain sections from mice that received retro-orbital injections were imaged with the BZ-X710 fluorescence microscope using a 4× objective lens and the automated tiling function.

For confocal imaging, objective lenses with magnifications of 10, 40, and 100× were used. The excitation laser wavelengths were 405, 488, 561, and 640 nm. For dendritic spine analysis, extended focus images were generated from *Z*-stacks that were acquired with SpinSR10 using 40 and 100× objective lenses and processed with the CellSens software (Olympus).

### Quantification of sparsity and brightness

GFP-positive and red fluorescent protein (RFP)-positive cells were manually counted in each specimen using Fiji (ImageJ), and the ratio of RFP-positive to GFP-positive cells (RFP/GFP) was defined as sparsity. Brightness was defined as the mean RFP fluorescence intensity of the soma.

### Quantification of Flpe-dependent penetrance in RFP-positive and RFP-negative cells

GFP-positive and RFP-positive cells in the hippocampal CA1 region were manually counted using an intensity threshold. As expression of Flpe could not be directly determined in our system, RFP-positive cells were considered Flpe-positive. The number of RFP-negative cells was estimated by counting Hoechst-stained nuclei within the analyzed hippocampal area as a surrogate measure.

### Quantification of Flpe-dependent elimination of EYFP signal

EYFP-positive and RFP-positive Purkinje cells in the cerebellum were manually counted using an intensity threshold. Purkinje cells were identified based on their characteristic morphology and laminar location. The loss of Flpe-dependent EYFP signal was quantified by calculating the proportion of EYFP/RFP double-positive cells among RFP-positive Purkinje cells.

### Statistics

No formal statistical analysis was performed.

## Results

### Flpe-dependent bright and sparse labeling of neurons

To enable the Cre-orthogonal labeling of neurons, we developed a Flpe-dependent two-component system that was based on the Supernova strategy (Fig. 1*A*, left; Mizuno et al., 2014; Luo et al., 2016). In this system, the driver expresses Flpe recombinase under control of the TRE promoter (Gossen and Bujard, 1992), and the amplifier, provided in excess, expresses tTA and red-fluorescent TurboRFP in a Flpe-dependent manner. Initially, the amplifier is inverted and flanked by two distinct flippase recognition target (FRT) sites, forming a flip-excision double–floxed inverted open reading frame (fDIO). We chose this design over the FRT-stop-FRT configuration because of its shorter length and tight expression control.

10.1523/ENEURO.0314-25.2026.f1-1 FIgure 1-1 Comparison of Flpe-dependent and Cre-dependent sparse labeling. A. Experimental condition of Flpe-dependent *in utero* electroporation. B. Tunable sparse labeling of P16 mouse cortical neurons with Flpe-dependent system (GFP, transfection marker; TurboRFP, sparse labeling; scale bar = 200 μm). C. Experimental condition of Cre-dependent *in utero* electroporation. D. Tunable but denser labeling of mouse P16 cortical neurons with Cre-dependent system (GFP, transfection marker; TurboRFP, sparse labeling; scale bar = 200 μm). E. Experimental condition of the AAV9 injection in the adult mouse hippocampus. F. Tunable sparse labeling with AAV9 in the hippocampal CA1 region at various driver/amplifier ratios (GFP, infection marker; TurboRFP, sparse labeling; scale bar = 200 μm). G. Sparsity of the cells labeled with AAV9 (left, RFP+/GFP + cells: 10⁵, 1.5%; 10⁴, 3.7%; 10³, 20%; geometric mean with individual data points; N = 2) and their brightness (right; box plots show the median and interquartile range, with whiskers indicating the full range; N = 2) Download FIgure 1-1, TIF file.

10.1523/ENEURO.0314-25.2026.f1-2 Figure 1-2 Inefficient sparse labeling by retro-orbital injection of PHP.eB AAV in the mouse central nervous system. A. Experimental condition of retro-orbital injection in adult mice. B. Inefficient labeling by retro-orbital PHP.eB AAV delivery 21 days after injection at a driver/amplifier ratio of 1: 10⁴ and driver-rich conditions (1:15 and 1:3 ratios; GFP, infection marker; TurboRFP, sparse labeling; scale bar = 2 mm). C. Rare RFP-positive cells in the hippocampal CA2 region and olfactory bulb (scale bar = 200 μm). Download Figure 1-2, TIF file.

Sparse labeling with this system relies on tTA-independent transcriptional activity of the TRE promoter. We used the TRE-tight promoter because its basal activity is higher than the latest TRE3G (Loew et al., 2010; Shima et al., 2016). Flpe recombinase that is expressed from the driver mediates irreversible inversion of the amplifier into the correct orientation. Once a single copy of the amplifier is recombined, it constitutively expresses tTA and TurboRFP (hereafter referred to as RFP) under the EF1α promoter. tTA binding to the driver then initiates a positive feedback loop that eventually converts the remaining amplifier copies to the on-state (Fig. 1*A*, right). As the entire process is independent of doxycycline, no doxycycline was administered.

We validated this system by labeling mouse cortical neurons via IUE (Saito and Nakatsuji, 2001). The driver and amplifier plasmids were mixed at various ratios and introduced into neuronal progenitors that lined the lateral ventricle on E15.5 (Fig. 1*B*). Consistent with previous Supernova-based approaches, our Flpe-dependent system achieved the sparse yet bright labeling of a subset of neurons (Fig. 1*C*, left). The labeled neurons exhibited sufficient fluorescence to visualize fine dendritic structures without immunostaining (Fig. 1*C*, right), and the labeling density was tunable by adjusting the driver/amplifier ratio.

We next compared the performance of our Flpe-dependent system with the previously reported Cre-dependent version (Extended Data Fig. 1-1*A–D*). Both systems enabled the tunable sparse labeling of cortical neurons, but the Cre-dependent system exhibited higher labeling efficiency, reflecting the well-established robustness of Cre-LoxP recombination (Zhao et al., 2023). Our results are consistent with previous findings that used a plasmid with an excisable stop sequence (Luo et al., 2016). The reduced labeling efficiency of our Flpe-based system may be attributable to the lower recombination efficiency by Flpe and its weaker expression compared with the codon-optimized variant, Flpo (Raymond and Soriano, 2007).

Although our Flpe-dependent system performed effectively in cortical neurons, IUE must be performed during development. To establish an easily adaptable ad hoc sparse labeling method in adult mice, we next attempted a retro-orbital injection of PHP.eB-packaged AAVs (Extended Data Fig. 1-2A). The retro-orbital injection of PHP.eB AAV, a blood–brain barrier-crossing variant of AAV, can efficiently transduce various types of neurons in adult C57BL/6J mice (Chan et al., 2017; Mathiesen et al., 2020). The same constructs that were used for IUE were packaged into PHP.eB capsids, and a mixture of the driver and amplifier viruses was injected in the retro-orbital sinus in adult mice.

Widespread EGFP expression confirmed successful transduction, but no RFP-positive cells were detected at a driver/amplifier ratio of 1:10^4^ (Extended Data Fig. 1-2*B*). Increasing the driver virus to 1:15 and 1:3 ratios should theoretically yield more RFP labeling. However, only a few neurons in the olfactory bulb and hippocampal CA2 region were labeled, and overall efficiency remained several orders of magnitude lower than with IUE (Extended Data Fig. 1-2*C*).

### Analyzing two-component labeling with a positive feedback process

Although our retro-orbital injection approach failed to label adult neurons sparsely, we noted that the labeled cells were enriched in the population with high EGFP expression. In our pilot experiments, we also found that the local hippocampal injection of the same driver-rich mixture resulted in extensive bright labeling around the injection site. Based on these observations, we hypothesized that the overall labeling efficiency was affected not only by the donor-to-amplifier ratio (D/A ratio) but also by total copy numbers of viral genomes in the cell, probably in a nonlinear manner. To address this issue, we analyzed a simplified model of our labeling method.

Here, we denote intracellular copy numbers of the driver and amplifier viruses as D and A, respectively (Fig. 1*D*, top). The total copy number, *N*, is the sum of D and A, where D≪A. The intracellular D/A ratio is expected to positively correlate with the predetermined ratio in the viral mixture. In the initial state, all amplifier genomes are in the off-state, and the intracellular concentration of Flpe recombinase (*C*_Flp_) is zero.

### Labeling efficiency is nonlinearly affected by total viral genomes per cell

Once a single copy of the amplifier undergoes two recombination events and is irreversibly fixed in the on-state, the remaining amplifier genomes are subsequently recombined through the positive feedback mechanism, resulting in their labeling (Fig. 1*A*). Therefore, it is important to characterize the probability of “first” irreversible recombination events to understand the overall labeling process. For each amplifier genome, the probability of undergoing irreversible recombination depends on the intracellular Flpe concentration and can be described as its increasing function, *F*(*C*_Flp_). In cells harboring multiple amplifier genomes, this probability scales with the number of amplifier copies (A), such that the overall probability can be approximated as *A* × *F*(*C*_Flp_) (Fig. 1*D*, bottom). Because amplifier genomes are dominant in the viral mixture, A closely approximates the total intracellular viral genome number, *N*. Consequently, even at a fixed D/A ratio, cells containing higher numbers of viral genomes exhibit an increased likelihood of being labeled, suggesting a nonlinear relationship.

Achieving optimal labeling, therefore, requires delivering a higher number of viral particles to target cells. Our labeling system inherently favors cells that harbor more viral genomes, thereby ensuring bright labeling.

### Neuronal labeling with tunable sparseness by local AAV injection

Based on these analyses, we next performed local stereotaxic AAV injections to deliver more viral particles (Fig. 1*E*). We injected the same PHP.eB AAV mixture in the hippocampus in adult wild-type mice.

As expected, a subset of CA1 excitatory neurons was brightly and sparsely labeled 21 d after the injection (Fig. 1*F*). The labeling density was tunable by adjusting the driver concentration (Fig. 1*G*, left). The mean brightness of the labeled cells increased with higher driver concentration; however, brightly labeled cells were also observed under low-driver conditions (Fig. 1*G*, right). The apparent increase in the mean brightness at higher driver concentrations was partly attributable to fluorescence contamination from the surrounding neurons. We also evaluated labeling using the AAV9 serotype. Labeling with AAV9 was tunable and the mean brightness increased with the driver concentration similarly (Extended Data Fig. 1-1*E*–*G*). The neurons labeled using our method exhibited sufficient fluorescence for morphological analysis. Even under the sparsest condition, dendritic spines were visualized without immunostaining (Fig. 1*H*).

The optimal driver/amplifier ratio for sparse labeling in the hippocampus was ∼1:10^4^ to 1:10^3^. In contrast, the retro-orbital injection could not label neurons even under most driver-rich conditions, highlighting the critical importance of viral copy numbers in target cells.

### Sparse labeling in the cerebellum in Cre-expressing mice

We next tested the applicability of this Flpe-dependent labeling system to other brain regions in Cre-expressing mice. The cerebellum, for which widely used and easily adaptable Cre-independent sparse labeling methods are limited, was selected as the target.

We injected the PHP.eB AAV mixture in the cerebellar lobule in adult L7-Cre mice, in which Cre recombinase is almost exclusively expressed in Purkinje cells, at a driver/amplifier ratio of 1:30 (Fig. 2*A*). Twenty-one days after the injection, GFP was widely expressed in Purkinje cells, and a subset of these cells (16%, 24/146, *N* = 2) was brightly labeled with RFP (Fig. 2*B*).

10.1523/ENEURO.0314-25.2026.f2-1 Figure 2-1 Sparse labeling in adult mice with a floxed genetic background. A. Experimental condition of local PHP.eB AAV injection in the cerebral cortex in adult Tsc1 floxed/floxed mice. B. Sparsely labeled cortical neurons 21 days after injection at a driver/amplifier ratio of 1:300 (GFP, infection marker; TurboRFP, sparse labeling; scale bar = 200 μm). C. Experimental condition of local PHP.eB AAV injection in the cerebellum in adult Tsc1 floxed/floxed mice. D. Sparsely labeled Purkinje cells 14 days after injection at a driver/amplifier ratio of 1:30 (GFP, infection marker; TurboRFP, sparse labeling; scale bar = 200 μm). E. Labeled fusiform interneuron in the granular layer (GFP, infection marker; TurboRFP, sparse labeling; scale bar = 200 μm). Download Figure 2-1, TIF file.

The entire dendritic arborization of each Purkinje cell and its dendritic spines were visualized without signal amplification (Fig. 2*C*,*D*). As this system is Cre-orthogonal, it functions in Cre-positive Purkinje cells of L7-Cre mice. Some granular layer interneurons were also labeled (Fig. 2*E*); however, no RFP-positive granule cells were observed, suggesting that viral tropism plays a role in cell-type specificity.

### Sparse labeling in other brain regions in a floxed genetic background

To take advantage of the Cre-orthogonal nature of our labeling strategy, we performed sparse neuronal labeling in a floxed genetic background. In our Flpe-dependent labeling, floxed genes remain intact because this system does not involve Cre recombinase.

The same AAV mixture was injected in the frontal cortex in Tsc1 floxed/floxed mice at a driver/amplifier ratio of 1:300 (Extended Data Fig. 2-1*A*). A subset of cortical neurons was brightly labeled 21 d after the injection (Extended Data Fig. 2-1*B*).

Similarly, in the cerebellar lobule in these mice, Purkinje cells and interneurons were brightly labeled 14 d after the injection (Extended Data Fig. 2-1*C–E*). These results demonstrate that the local injection of PHP.eB AAVs enables the bright and sparse labeling of neuronal subsets in the hippocampus, cortex, and cerebellum across various genetic backgrounds.

### Sparse labeling with Cre-dependent gene knock-out

We next evaluated the compatibility of this system with Cre-dependent gene knock-out. We coinjected the PHP.eB AAV labeling mixture with a virus that expressed either GFP or Cre-GFP in the hippocampus in Tsc1 floxed/floxed mice at a driver/amplifier ratio of 1:500 (Fig. 3*A*).

A subset of neurons was brightly labeled with RFP 21 d after the injection (Fig. 3*B*). The immunofluorescence intensity against Tsc1 protein decreased in Cre-expressing cells compared with the contralateral control (Fig. 3*C*). Our Cre-orthogonal labeling system allowed direct morphological comparisons between control and Tsc1-knock-out neurons within the same subject.

### Genetic manipulation of labeled cells via coinfection with the third virus

We tested whether the labeled neurons could be genetically manipulated via coinfection with a third virus. We injected the PHP.eB AAV mixture, together with a Flpe-dependent hM4Di-expressing virus, in the hippocampus in wild-type mice at a 1:500 driver/amplifier ratio (Fig. 3*D*).

Flpe recombinase that was expressed in RFP-positive cells recombined the third viral genome, resulting in the expression of hM4Di-EGFP in 68% (17/25 RFP-positive cells, 20 sections, *N* = 3) of the RFP labeled cells (Fig. 3*E*,*F*). We estimated the penetrance among RFP-negative cells to be 0.032% (1/3,091 nuclei, 20 sections, *N* = 3), using Hoechst-stained nuclei as a surrogate for the number of RFP-negative cells. Under our experimental conditions, 94% of GFP-positive cells (17/18 GFP-positive cells, 20 sections, *N* = 3) were RFP-positive. Although the penetrance was incomplete, these findings indicated that our Flpe-dependent sparse labeling system allowed the functional control of labeled neurons.

Finally, we directly tested whether the labeled neurons express Flpe recombinase. We injected PHP.eB AAV mixture containing Con/Foff 2.0-EYFP virus into the cerebellum of L7-Cre mice (Fig. 3*G*). Upon infection, EYFP is expressed in Cre-positive cells, but its expression is turned off in the presence of Flpe. As expected, most Purkinje cells were labeled with EYFP. Notably, EYFP signals were completely absent from RFP-positive Purkinje cells (Fig. 3*H*,*I*; 0%, 0/11 RFP-positive cells, eight sections, *N* = 2), indicating that the labeled neurons indeed expressed Flpe recombinase.

## Discussion

Sparse labeling techniques are essential for analyzing the structure and function of the central nervous system. Easily adaptable ad hoc methods, which are particularly applicable to Cre-dependent genetic backgrounds, have long been sought. In the present study, we demonstrated that our AAV-based, Flpe-dependent system enables the bright and sparse labeling of a subset of neurons across various brain regions in adult mice.

### Comparison with existing sparse labeling methods

To visualize the morphology of individual neurons in densely packed neural tissue, we have developed various elaborate labeling methods. Multicomponent systems, such as Brainbow and Tetbow techniques, label neurons in multiple colors, allowing the discrimination of adjacent cells based on differences in color hue (Livet et al., 2007; Sakaguchi et al., 2018; Leiwe et al., 2024).

Other approaches achieve sparse labeling using the Thy-1 promoter or its variant. Promoter-assisted sparse-neuron multiple-gene labeling using IUE (PASME) employs a shortened version of the Thy-1 promoter, which is compatible with AAV vectors. However, its use is limited in cells that express Thy-1 (Ako et al., 2011). BATTLE-1, BATTLE-2, and BATTLE-2.1 systems utilize multiple driver lentiviruses (Kohara et al., 2020). Each driver virus expresses a different recombinase whose coding sequence can be excised by another recombinase. These systems require two injections, first the driver and then the amplifier, but their mechanism of action remains particularly interesting.

Currently, two types of AAV-based sparse labeling strategies are commonly used. The first strategy involves either a PHP.eB AAV injection in adult mice or an intraventricular AAV injection during the early postnatal period. In these methods, low-density viral infection limits the number of viral genomes per cell, leading to insufficient fluorescence signals. Furthermore, the experimenter has little control over physical spread of the virus because viral particles are distributed via the bloodstream or cerebrospinal fluid.

The second strategy employs two components: a driver and an amplifier. The driver expresses a recombinase or tTA, and the amplifier expresses fluorescent proteins in a driver-dependent manner. These methods achieve brighter labeling because of the higher copy number of the amplifier virus, but they often result in variable fluorescence intensity among cells (Chan et al., 2017).

Our analysis predicts that labeling probability in a simple two-component system without positive feedback depends solely on the driver amount. In contrast, a two-component system with positive feedback, such as Supernova, preferentially labels cells that receive more viral genomes, providing consistent brightness. In a previous study, the authors demonstrated that a local injection of a Cre-dependent Supernova AAV pair in the cerebral cortex in Postnatal Day 10–13 mouse pups resulted in brightly and sparsely labeled cortical neurons (Luo et al., 2016). They speculated that a Flpe-dependent version could be applied to Cre-expressing or floxed mice. We confirmed that our Flpe-dependent viral system functions well in such genetic backgrounds and is fully compatible with Cre-dependent tools.

### Comparison with a related Supernova-based labeling strategy

Lin and colleagues previously developed a Supernova-based labeling system in which TRE-driven Flpo recombinase activates a TRE-driven amplifier exclusively in Cre-positive cells (Lin et al., 2018). Although practical and elaborately designed, their method is optimized for labeling Cre-expressing populations.

Our method differs in three important ways. First, our system is Cre-orthogonal and thus compatible with Cre-dependent tools. It allows the labeling of both Cre-expressing and nonexpressing cells. Cre-expressing cells can be distinguished by coinfection with a Cre-dependent virus that expresses fluorescent proteins. Second, in their system, the initial Flpo-mediated recombination places the amplifier in a standby configuration rather than directly switching it to the on-state. A subsequent stochastic event is required to initiate the positive feedback loop, whereas our system immediately transitions to the on-state upon Flpe recombination. Third, their system sometimes yields Flpo-positive unlabeled cells. These “cryptic” cells can be problematic if one attempts downstream genetic manipulation using a Flpo-dependent third virus.

### Estimation of sparse labeling efficiency

Our analysis demonstrated that labeling efficiency depends on both the driver/amplifier ratio and infection efficiency. This finding provides valuable guidance for optimizing labeling sparseness. For example, brain regions with higher viral transduction efficiency may require fewer driver genomes to obtain optimal labeling.

Recently, Maturana and colleagues modeled the infection process after local PHP.eB AAV injection as a Poisson distribution, estimating the number of incoming viral genomes (Maturana et al., 2023). Based on their analysis, the lower bound of viral genomes that are delivered to mouse cortical and cerebellar neurons was estimated to be ∼20. Applying their methodology to previously published data from a three-color PHP.eB AAV injection study (Chan et al., 2017), we estimated that the retro-orbital administration of 2 × 10^11^ viral genomes per animal results in fewer than eight and four genomes that are delivered per cortical and cerebellar neuron, respectively. This viral dose is comparable to our experiments.

These estimates enable the simulation of viral genome distribution, which is particularly informative in coinfection scenarios that involve multiple AAVs. Notably, however, given the markedly different labeling sparseness that is observed between intraretro-orbital and local injection methods in our experiments, a subset of neurons likely receives substantially more viral genomes than the estimated lower bound following local injection.

### Technical limitations of the system

Our AAV-based labeling system is remarkably simple—inject, wait, and observe in your favorite mice whenever you want. This system is compatible with immunohistochemistry. It can thus be used to analyze the morphology of neuron subtypes that are distinguished by specific protein markers. Tissue-specific promoters can also be combined to visualize specific cell types (Graybuck et al., 2021; Mich et al., 2021; Ben-Simon et al., 2025; Furlanis et al., 2025).

Although we used the PHP.eB and AAV9 capsid in the present study, other AAV capsids may allow the targeting of different cell types (Brown et al., 2021). Additionally, because the system does not rely on species-specific components, it has potential applications in other species, including organoids, provided that viral delivery is feasible.

Nonetheless, several technical limitations must be taken into account. First, the optimal driver/amplifier ratio needs to be empirically determined for each brain region. Overall labeling density is influenced by three main factors: infection efficiency, transcriptional activity of the driver and amplifier promoters, and leakiness of the TRE promoter in targeted cells. For example, optimal cerebellar labeling required much more driver virus than hippocampal labeling. However, granule cells remained unlabeled. This suggests that viral tropism and cell-intrinsic promoter activity contribute to cell-type specificity. Testing multiple AAV serotypes may be necessary to identify optimal conditions. Second, we delivered high-titer AAVs via local injection; thus, a spatial gradient in infection is unavoidable. We observed more RFP-positive cells near the injection center, and careful titration was necessary to achieve an optimal labeling density that is slightly off-center to avoid confounding effects from local overinfection. Third, this method is unsuitable for labeling dividing cells. Daughter cells from labeled progenitors have fewer amplifier genomes per cell and may not inherit the Flpe-expressing genome. Fourth, immune responses must be considered. Both the injection procedure and AAV infection can trigger inflammation. Sufficient recovery time (>1 week) should be allowed before analysis to avoid inflammatory effects. Fifth, potential toxicity from the high-level expression of tTA and Flpe is a concern. Although Cre toxicity is well documented (Loonstra et al., 2001), the prolonged overexpression of tTA and Flpe via positive feedback may also impact cell viability (Gallia and Khalili, 1998).

### Future direction and possible applications of the system

Despite these limitations, our AAV-based, Cre-orthogonal, Flpe-dependent labeling system enables the bright and sparse labeling of neurons across various brain regions. The system can be readily implemented in existing experimental frameworks and is compatible with both immunohistochemistry and Cre-dependent genetic tools. Furthermore, it supports diverse downstream applications. Because RFP-positive cells express both tTA and Flpe, effectors that are controlled by TRE, Flpe, or both can be selectively targeted to these cells (Fig. 3*E*). Labeled cells can be manipulated simply by coinfecting a third virus. Notably, each neuron is estimated to receive >20 viral genomes following a local PHP.eB AAV injection (Maturana et al., 2023). This supports the feasibility of downstream manipulation using multiple AAVs. Such manipulations may include employing viruses that encode optogenetic or chemogenetic tools to selectively modulate the activity of labeled cells. Additionally, other recombinases, such as Cre, genome-editing systems (e.g., CRISPR), and RNAi, can be combined with the labeling to enable simultaneous genetic modifications. Furthermore, a TVA-expressing virus can be used to prepare starter cells for circuit tracing with pseudotyped rabies virus (Wickersham et al., 2007). However, minimizing unintended expression will require further refinement (Fischer et al., 2019).

The intense fluorescence of labeled cells provides practical advantages. Entire projection trajectories from labeled neurons can be visualized in situ using tissue-clearing techniques (Susaki et al., 2014; Hama et al., 2015; Ke et al., 2016). In future applications, this system could be adapted for in vivo structural imaging.

Compared with specialized transgenic mouse lines, our approach is easier to integrate into existing experimental systems. It also allows broad application to a wide range of Cre-expressing and floxed animals, as well as various disease models. Although TurboRFP was used in the present study, amplifiers that encode alternative fluorescent proteins or that lack fluorescent proteins altogether produced comparable results. Finally, because this method is compatible with well-established Cre–dependent labeling, combining it with our system will allow dual-color sparse labeling that is suitable for in situ, side-by-side comparisons within the same subject.

## Synthesis

Reviewing Editor: Sam Golden, The University of Washington Decisions are customarily a result of the Reviewing Editor and the peer reviewers coming together and discussing their recommendations until a consensus is reached. When revisions are invited, a fact-based synthesis statement explaining their decision and outlining what is needed to prepare a revision will be listed below. The following reviewer(s) agreed to reveal their identity: Matthew Pomrenze.

This manuscript was reviewed by two experts in the field, and both felt that this is an interesting and simple Flp recombinase-based system for sparse labeling of neuronal populations. The approach is cre-orthogonal, enabling sparse labeling in genetic models subjected to other cre-loxP recombinations. The implications of this technique are sound and could be widely adopted. However, the evidence included relies heavily on observational accounts and might seem anecdotical at times. This is accompanied by a poorly laid out mathematical model, extremely generic and lacking fit data, without a clear purpose. Especially relevant for a tool development paper, the sparsity-concentration relationship is not well characterized, making the adoption of the TRE-tTA-TurboRFP system by other groups extremely difficult. The specific major and minor concerns are as follows:

Major Concerns

Both reviewers felt the manuscript requires formal, quantitative and replicable evidence, as detailed:

1. Several key claims are not supported by quantitative evidence. Characterizing the relative sparsity achieved by increasing relative concentrations of the viral cocktail is crucial for others to use this tool. This is missing from the manuscript. More concentrations are needed to properly characterize this relationship. Quantitative claims (i.e., dose-dependent effects, degree of sparsity, specificity of infection) must be backed up by quantitative evidence. Authors claim that "Labeling efficiency and brightness are affected by total viral genomes per cell", but they do not compare labeling efficiency and brightness across relative viral concentrations. Authors also claim that "The initial number of amplifier genomes positively correlates with the total intracellular viral genome count" without providing any correlation of observed or model-simulated data. More examples: "a subset of these cells was brightly labeled with RFP as intended" - what percentage of Purkinje cells were infected? "Because this system is Cre-orthogonal, it functions regardless of the cells" Cre-expressing status" - what percent of cre+ vs cre- cells expressed RFP? "Flpe recombinase that was expressed in RFP-positive cells recombined the third viral genome, resulting in the expression of hM4D(Gi)-EGFP" What is the relative penetrance of hM4D(Gi) in Flpe+ vs Flpe- cells? "We estimated the expected number of the "first" irreversible recombination events to characterize the entire labeling process" - Where is that number?

Some basic parameters required to use the tool are not properly characterized, as noted by both reviewers:

3. The authors use a tetracycline response element (TRE) to drive Flp expression, which controls reporter transgene expression. Spontaneous leakage of this promoter is what drives initial Flp expression, which goes on to self-amplify. But there is no mention of tetracycline administration to the subjects, nor any advice given for how to use tetracycline with this system. As is, the method is confusing because tetracycline and TRE are commonly used together. The authors need to explain in the methods whether tetracycline is used here or not, and if it is, it must be explained clearly for the future users of this technology.

4. Use of the PHP.eB AAV is logical to test viral infection efficiency as a function of distance from target cells. However, it seems an odd choice to infuse this serotype into the brain, as this is atypical for majority of neuroscience studies using AAVs. The authors should validate this sparse labeling system in a serotype that neuroscientists use regularly, such as AAV5, AAV8, or AAV9. Without that data, future users of these constructs will need to beta test them if they have a preferred serotype. Knowing that this system works just as well (or less well) in typical AAV serotypes will go a long way in determining how generic and applicable this labeling strategy is.

5. Figure 2 shows very nice proof of concept data that this strategy leads to robust sparse labeling. However, the use of the L7-Cre line seems unnecessary. As it reads, injecting the novel constructs into the Cre line doesn't quite demonstrate orthogonality to Cre. A better strategy might be to cocktail the new viruses with a Cre-ON-Flp-OFF GFP, which would be predicted to infect only L7 cells and no neurons labeled with TurboRFP. This AAV is commercially available (https://www.addgene.org/137162/), and the authors should strongly consider this validation approach.

The mathematical model was found to lack details and focus:

6. The authors claim that they created a mathematical model to describe their self-amplifying method. Yet, the only formula included is "A×F(CFlp)", which describes a simple linear relationship between 3 variables. Where is the formula (or formulae) of the model?

From this model, the authors presumably conclude that "the expected number of the first irreversible recombination events is proportional to the initial number of amplifier genomes" This follows common logic and does not require mathematical symbols.

7. It is stated that Fig 1D illustrates the positive feedback sustaining the self-amplification between tTA and Flpe but this is poorly laid out graphically. Logically, there cannot be a positive feedback loop if no arrows point from Aon to Aint1-2. I recommend the authors remove this section entirely.

Images need to be of better quality/number to clearly demonstrate findings:

8. When shown, fine dendritic structure images are too decontextualized. For the reader, it is impossible to ascertain whether these images (Fig3D, for instance) actually comes from a cre+ cell. Images with zoomed in insets should be used to simultaneously illustrate the sparsity, specificity and cre-independence of the proposed method.

Minor comments

1. Please rewrite paragraph 67-73 for clarity. It was difficult for the reader to understand.

2. Fig3A: Why did the authors use 1:500 and not 1:10^4 or 3?

3. Panel B from Extended data Fig2-1 does not show spare labeling. It would be challenging to separately reconstruct the dendritic structure of each of those cells.

4. Authors state that there is no ad hoc method for sparse labeling in the cerebellum, compared to other brain structures. Why is that? Could you provide some references supporting this?
