# Supplementary figures and images for: AAV-Based Bright and Sparse Labeling of Versatile Neurons Adaptable in Cre-Dependent Genetic Backgrounds
Source: eNeuro. 2026 Mar 25;13(3):ENEURO.0314-25.2026. doi: 10.1523/ENEURO.0314-25.2026 (PMC13064814; doi:10.1523/ENEURO.0314-25.2026)

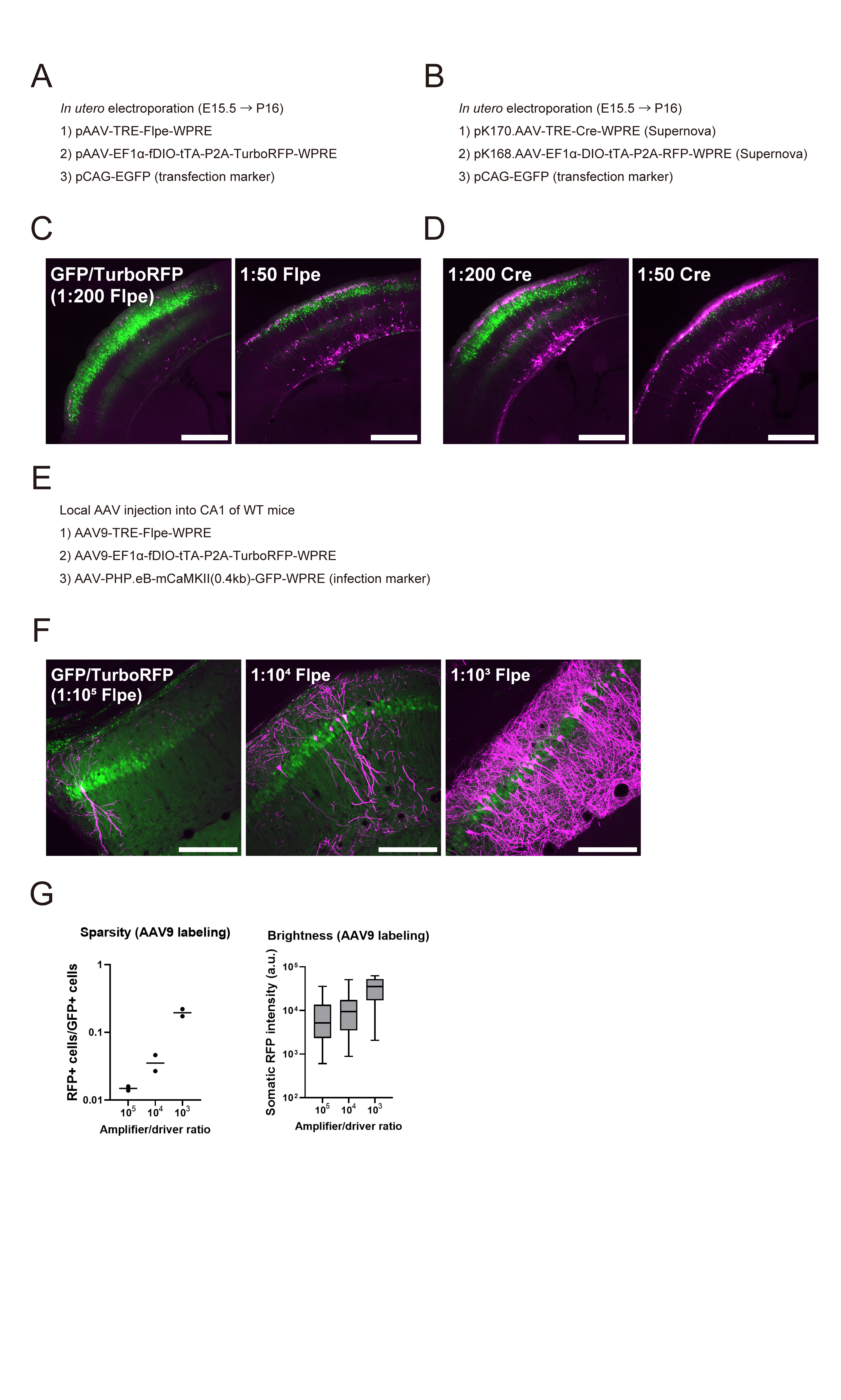

Supplement: FIgure 1-1 — Comparison of Flpe-dependent and Cre-dependent sparse labeling. A. Experimental condition of Flpe-dependent in utero electroporation. B. Tunable sparse labeling of P16 mouse cortical neurons with Flpe-dependent system (GFP, transfection marker; TurboRFP, sparse labeling; scale bar = 200 μm). C. Experimental condition of Cre-dependent in utero electroporation. D. Tunable but denser labeling of mouse P16 cortical neurons with Cre-dependent system (GFP, transfection marker; TurboRFP, sparse labeling; scale bar = 200 μm). E. Experimental condition of the AAV9 injection in the adult mouse hippocampus. F. Tunable sparse labeling with AAV9 in the hippocampal CA1 region at various driver/amplifier ratios (GFP, infection marker; TurboRFP, sparse labeling; scale bar = 200 μm). G. Sparsity of the cells labeled with AAV9 (left, RFP+/GFP + cells: 10⁵, 1.5%; 10⁴, 3.7%; 10³, 20%; geometric mean with individual data points; N = 2) and their brightness (right; box plots show the median and interquartile range, with whiskers indicating the full range; N = 2) Download FIgure 1-1, TIF file. [file eneuro-13-ENEURO.0314-25.2026-s002.tif]

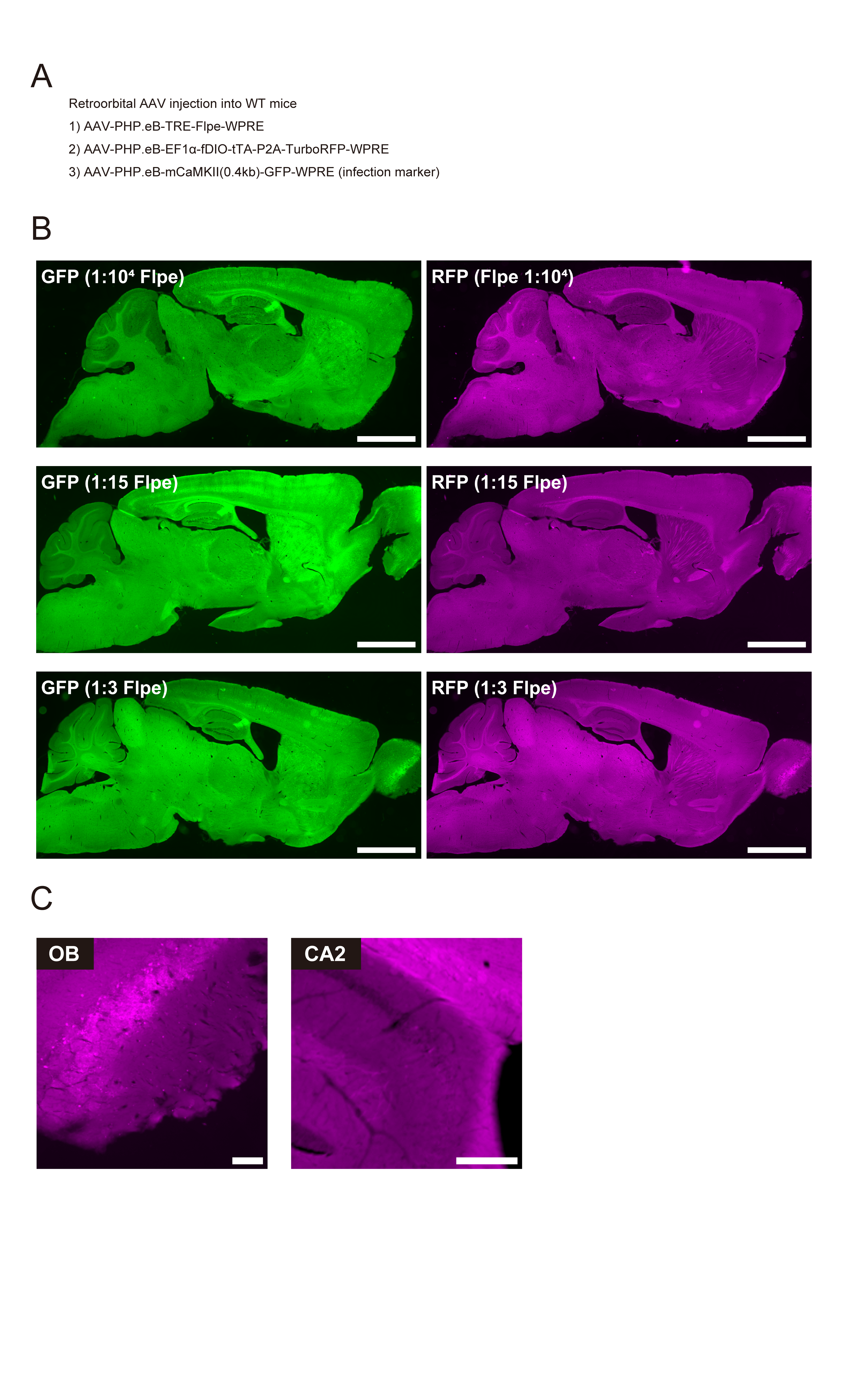

Supplement: Figure 1-2 — Inefficient sparse labeling by retro-orbital injection of PHP.eB AAV in the mouse central nervous system. A. Experimental condition of retro-orbital injection in adult mice. B. Inefficient labeling by retro-orbital PHP.eB AAV delivery 21 days after injection at a driver/amplifier ratio of 1: 10⁴ and driver-rich conditions (1:15 and 1:3 ratios; GFP, infection marker; TurboRFP, sparse labeling; scale bar = 2 mm). C. Rare RFP-positive cells in the hippocampal CA2 region and olfactory bulb (scale bar = 200 μm). Download Figure 1-2, TIF file. [file eneuro-13-ENEURO.0314-25.2026-s003.tif]

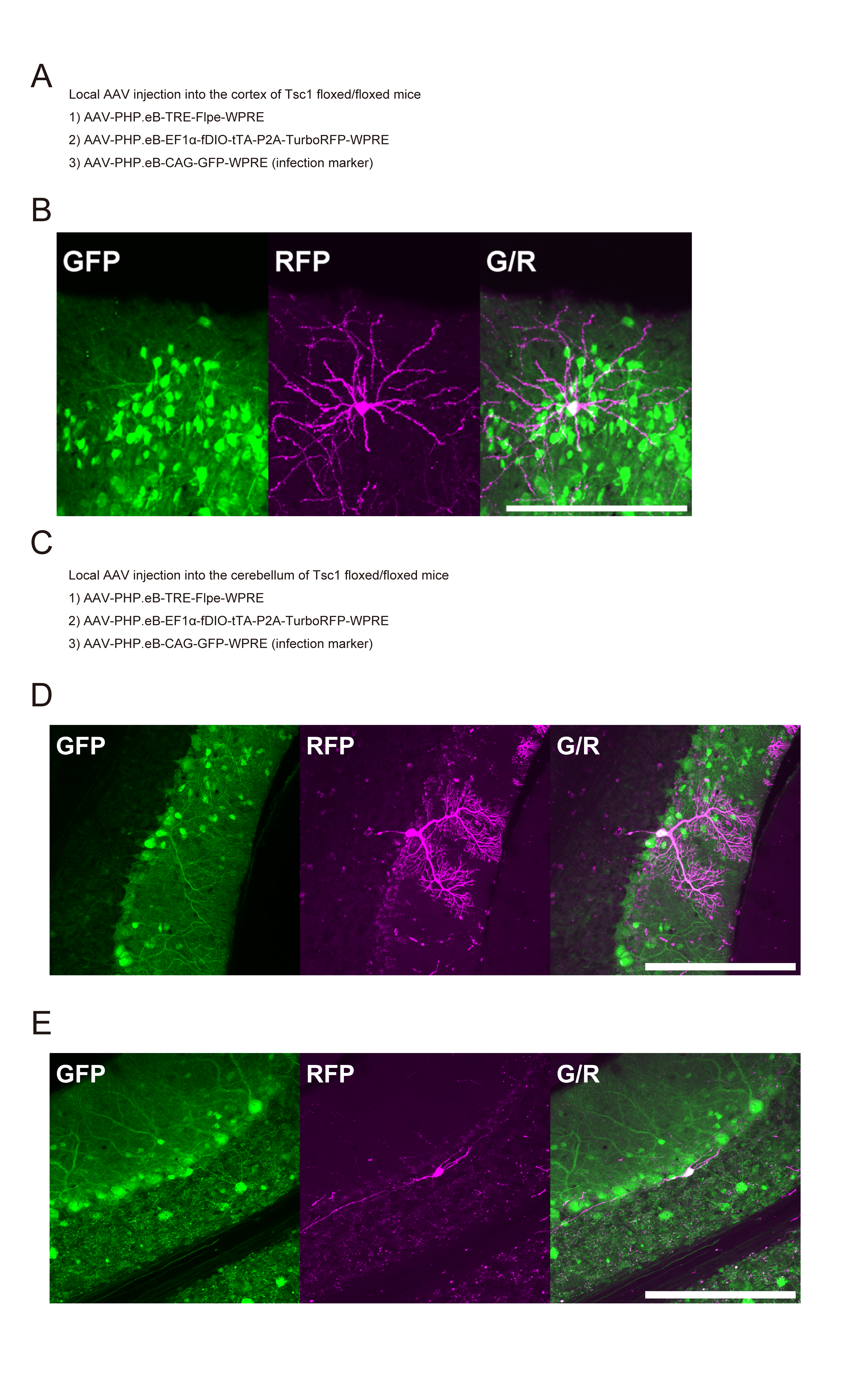

Supplement: Figure 2-1 — Sparse labeling in adult mice with a floxed genetic background. A. Experimental condition of local PHP.eB AAV injection in the cerebral cortex in adult Tsc1 floxed/floxed mice. B. Sparsely labeled cortical neurons 21 days after injection at a driver/amplifier ratio of 1:300 (GFP, infection marker; TurboRFP, sparse labeling; scale bar = 200 μm). C. Experimental condition of local PHP.eB AAV injection in the cerebellum in adult Tsc1 floxed/floxed mice. D. Sparsely labeled Purkinje cells 14 days after injection at a driver/amplifier ratio of 1:30 (GFP, infection marker; TurboRFP, sparse labeling; scale bar = 200 μm). E. Labeled fusiform interneuron in the granular layer (GFP, infection marker; TurboRFP, sparse labeling; scale bar = 200 μm). Download Figure 2-1, TIF file. [file eneuro-13-ENEURO.0314-25.2026-s004.tif]
